# A Novel Missense Variant in Actin Binding Domain of *MYH7* Is Associated With Left Ventricular Noncompaction

**DOI:** 10.3389/fcvm.2022.839862

**Published:** 2022-04-08

**Authors:** Mahdi Hesaraki, Ugur Bora, Sara Pahlavan, Najmeh Salehi, Seyed Ahmad Mousavi, Maryam Barekat, Seyed Javad Rasouli, Hossein Baharvand, Gunes Ozhan, Mehdi Totonchi

**Affiliations:** ^1^Department of Developmental Biology, School of Basic Sciences and Advanced Technologies in Biology, University of Science and Culture, Tehran, Iran; ^2^Department of Stem Cells and Developmental Biology, Cell Science Research Center, Royan Institute for Stem Cell Biology and Technology, ACECR, Tehran, Iran; ^3^Izmir Biomedicine and Genome Center (IBG), Dokuz Eylul University Health, Izmir, Turkey; ^4^Izmir International Biomedicine and Genome Institute (IBG-Izmir), Dokuz Eylul University, Izmir, Turkey; ^5^Department of Genetics, Reproductive Biomedicine Research Center, Royan Institute for Reproductive Biomedicine, ACECR, Tehran, Iran; ^6^School of Biological Science, Institute for Research in Fundamental Sciences (IPM), Tehran, Iran; ^7^Department of Regenerative Medicine, Cell Science Research Center, Royan Institute for Stem Cell Biology and Technology, ACECR, Tehran, Iran; ^8^Department of Tissue Morphogenesis, Max Planck Institute for Molecular Biomedicine, Münster, Germany

**Keywords:** cardiomyopathy, LVNC, WES, myosin heavy chain 7, zebrafish

## Abstract

Cardiomyopathies are a group of common heart disorders that affect numerous people worldwide. Left ventricular non-compaction (LVNC) is a structural disorder of the ventricular wall, categorized as a type of cardiomyopathy that mostly caused by genetic disorders. Genetic variations are underlying causes of developmental deformation of the heart wall and the resultant contractile insufficiency. Here, we investigated a family with several affected members exhibiting LVNC phenotype. By whole-exome sequencing (WES) of three affected members, we identified a novel heterozygous missense variant (c.1963C>A:p.Leu655Met) in the gene encoding myosin heavy chain 7 (*MYH7*). This gene is evolutionary conserved among different organisms. We identified *MYH7* as a highly enriched myosin, compared to other types of myosin heavy chains, in skeletal and cardiac muscles. Furthermore, *MYH7* was among a few classes of *MYH* in mouse heart that highly expresses from early embryonic to adult stages. *In silico* predictions showed an altered actin-myosin binding, resulting in weaker binding energy that can cause LVNC. Moreover, CRISPR/Cas9 mediated *MYH7* knockout in zebrafish caused impaired cardiovascular development. Altogether, these findings provide the first evidence for involvement of p.Leu655Met missense variant in the incidence of LVNC, most probably through actin-myosin binding defects during ventricular wall morphogenesis.

## Introduction

Left ventricular non-compaction (LVNC) is a structural abnormality of the cardiac muscle that presents in the ventricular wall, especially in the left ventricle, leading to extended trabeculation (1), and ultimately resulting in cardiomyopathies. LVNC follows an autosomal dominant inheritance pattern (2, 3), and could develop into both hypertrophic cardiomyopathy (HCM) and dilated cardiomyopathy (DCM) (4). Often, a genetic cause leads to LVNC that can be traced in a family which affects only the heart. The age of diagnosis varies between individuals, and ranges from birth to late adulthood. The precise cellular and molecular mechanisms of some LVNC subtypes are presently unknown. However, the time of onset and severity of the disease might be influenced by genetic conditions or developmental events such as tension heterogeneity-induced cardiac trabeculation (5, 6). Previously, mutations in various sarcomeric genes such as *MYH7* (OMIM# 160760), *MYBPC3* (OMIM# 600958), or *TNNT2* (OMIM# 1910450) were associated to LVNC and introduced as major causes for this phenotype (4, 7). However, the myocardial wall morphogenesis should be also taken into account which has been shown to be tightly regulated by several signaling pathways, including TGFβ signaling (5, 8), NOTCH signaling (5, 9), Neuregulin/Erbb signaling (5, 8), and FGF signaling (8). Given the importance of sarcomeric structure and its function in cardiac contractility, any defects in sarcomeric components could result in cardiomyopathy leading to abnormalities in cardiovascular system (10). However, pathogenic variations in sarcomeric genes specifically actin and myosin, might also influence cardiac wall development (5) and cause cardiomyopathy.

The *MYH7* gene known as the myosin heavy chain beta (MHC-β) which is classified as a type I fiber and also known as slow-twitch fiber (11). This protein is a critical component of sarcomeric structure and has several interactions with other key proteins such as actin TNN, MYBPC3 (12, 13).

Here, we report a novel heterozygous missense variant in the *MYH7* gene in an Iranian family with a high prevalence of LVNC. *In silico* analysis was performed to check the availability and developmental presence of cardiac *MYH7* transcripts. Moreover, the effect of this missense variation in *MYH7*-actin binding was modeled which suggests its potential impact on the development of LVNC in this family. Using CRISPR/Cas9 genome editing system, a zebrafish model of *MYH7* was generated which showed developmental defects in early stages of heart formation. To our knowledge, this is the first *MYH7* knockout model of zebrafish that also has an apparent cardiovascular malformation.

## Materials and Methods

### Ethical Statement

The Institutional Review Board of the Royan Institute Research Center and the Royan Ethics Committee (Tehran, Iran) approved this study. Written informed consent was obtained from all members of this family or their guardians. Zebrafish are maintained in accordance with the guidelines of the Izmir Biomedicine and Genome Center's Animal Care and Use Committee. All animal experiments were performed with the approval of the Animal Experiments Local Ethics Committee of Izmir Biomedicine and Genome Center (IBG-AELEC). All methods were performed in accordance with the declaration of Helsinki 1975.

### Subjects

This study was performed between 2018 and 2019 in Iran and comprised 20 genetically dependent members diagnosed with LVNC, who were between 12 and 53 years of age. The patients were diagnosed at the Royan Institute Cardiac Clinic (Tehran-Iran) by physical examination, electrocardiogram (ECG), and echocardiogram (Echo) assessments. All affected members had severe deep recesses and extended trabeculations in their left ventricular myocardia, and had normal heart valve function without additional symptoms related to other disorders (Table 1).

**Table 1:** Overview of clinical features of LVNC in the affected family members.

**Individual identifier**	**1**	**2**	**3**	**4**	**5**	**6**	**7**	**8**
Affected individual	II-2	II-4	II-8	II-6	II-10	III-8	III-9	III-10
Gender	M	M	M	F	F	M	M	M
Age at last examination (years)	54	50	43	53	56	17	12	Died at 12
Gene	*MYH7*	*MYH7*	*MYH7*	*MYH7*	*MYH7*	*MYH7*	*MYH7*	unknown
Chromosome change (Hg19)	c.C1963A	c.C1963A	c.C1963A	c.C1963A	c.C1963A	c.C1963A	c.C1963A	unknown
Protein change	p.L655M	p.L655M	p.L655M	p.L655M	p.L655M	p.L655M	p.L655M	unknown
Mutation type	Missense	Missense	Missense	Missense	Missense	Missense	Missense	-
Allele type	Hetero	Hetero	Hetero	Hetero	Hetero	Hetero	Hetero	unknown
Confirmation method	WES and S.S	WES and S.S	WES and S.S	S.S	S.S	S.S	S.S	-
ECG	Abnormal	Abnormal	Abnormal	Abnormal	Abnormal	Abnormal	Abnormal	Abnormal
Echo	LVNC	LVNC	LVNC	LVNC	LVNC	LVNC	LVNC	LVNC
MRI	LVNC	LVNC	LVNC	-	-	LVNC	LVNC	-
Other	Diabetes, hypertension		Dizziness, hypertension, peripheral edema, dyspnea					

*M, Male; F: Female; LVNC, Left ventricular non-compaction cardiomyopathy; Hetero, Heterozygous; ECG, Electrocardiography; Echo, Echocardiography; MRI, Magnetic resonance imaging; WES, Whole-exome sequencing; SS, Sanger sequencing*.

### Genomic Characterization

Peripheral blood samples were obtained for molecular analysis from all members of the family (*n* = 20). All genomic DNA was isolated by the salting out DNA extraction method and quantified with a NanoDrop 1000 Spectrophotometer (Thermo Fisher). The inheritance of LVNC in this pedigree follows an autosomal dominant pattern. Thus, we included 3 brothers of second generation in the WES study and skipped others. WES was performed using the Illumina NovaSeq platform and generated paired-end reads of 150 base pairs with an average coverage of 100X. Reads were aligned to genome reference consortium human 19 (GrCh37) with the Burrows-Wheeler Aligner (BWA, V.0.7.17) (14). The duplicated reads were removed by the Sequencing Picard tool. The Genome Analysis Toolkit (GATK, V4), followed by the best practice pipeline (15), was used for preprocessing the alignment results, detection and removal of duplicate reads, base recalibration, and variant calling. The VCF Filter toolkit was used for post-processing the variant calling results and variants that had a minimum depth of 12, mapping quality of 40, and quality of 30 were retained. The wANNOVAR website (http://wannovar.wglab.org/) was used for annotations (16). After the annotation process, all synonymous variations and those with ExAC frequency >0.01 were filtered by PolyPhen-2 (http://genetics.bwh.harvard.edu/pph2/), the prediction algorithms. The sorting intolerant from tolerant (SIFT) (http://sift.bii.a-star.edu.sg/) and MutationTaster (http://www.mutationtaster.org) were used to evaluate candidate variations.

### Segregation Analysis and Sanger Sequencing

DNA samples from 20 members of the family were screened for the *MYH7* candidate variant via the semi-nested PCR and Sanger sequencing methods. The primers used in this study were: F1: 5-ACTGTCGTGGGCTTGTATCA-3; F2: 5-CTGTCTCCTTGGTGCATTCG-3; and R1: 5-TGGTGGTAGGTAGGGAGATG-3. All PCRs were carried out in 25 μl volumes that consisted of a 12.5 μl PCR master mix, ~100 ng DNA, 1 μL of each primer (10 μM), and ~15 μL double distilled water (DDW). Cycling conditions involved denaturation (94 °C, 10 min) followed by 30 cycles of 94°C for 40 s, 54/57°C for 35 s, 72°C for 45/35 s, and a final extension at 72°C for 5 min. Finally, the sequencing results were aligned to the reference sequence in the CLC Main Workbench 5.5 software.

### *MYH7* Expression Patterns

The expressions of the MYH protein classes in different tissues were compared using public databases. The BioGPS (http://biogps.org/) expression database was the best to evaluate all *MYHs* expression patterns in the human tissues. In the next step, the expression pattern of *MYH7* during developmental stages of the mouse heart (E10.5-Old) was checked in a high-throughput expression data set (GEO: GSE93271, expression profiling by array,) and a *p* < 0.05 was considered as significantly different in the signal intensity (17). Then, the expression level of *MYH7* (also known as ventricle myosin heavy chain like (*vmhcl*) as a zebrafish homolog of *MYH7* in human) was checked in zebrafish heart at different developmental stages using a high throughput expression dataset (GEO: GSE120236, RNA-sequencing) (18) and a *p* < 0.05 on normalized counts was considered as significantly different. All expression analyses were performed using pheatmap package in RStudio.

### Structural Analysis of *MYH7*

The 3D structure of *MYH7* was retrieved from the RCSB database (PDB ID:5TBY) for structural analysis. The p.Leu655Met mutation of the protein structure was induced by the psfgen plugin of VMD 1.9.3 (19). Both the wild type and mutant structures were minimized for 20 000 steps by the conjugate gradient method. Minimizations were done in NAMD 2.12 package (20) with CHARMM27 force field (21). The HADDOCK2.2 web server, which is an information-driven flexible docking approach (22, 23), was used to evaluate the effect of the p.Leu655Met mutation on the interaction between *MYH7* and actin. The actin binding regions in *MYH7* (655 to 677 and 757 to 771 residues) were set as the “active residues.” The surface neighbors of the active residues were selected as the “passive residues” that were automatically determined by HADDOCK2.2. The actin 3D structure and binding residues in interaction with *MYH7* (residues of 3, 23, 24, 28, 30, 146–148, 333–345, 348, 350 from PDB ID of 4A7F) and the common binding site for actin-binding proteins on the actin surface (residues of 143, 144, 146, 148, 168, 341, 345, 346, 349, 351, 355) (24) were set as the “active residues” in interaction with *MYH7*. All other HADDOCK settings were kept at the default values. The docking results were ranked based on the HADDOCK score, which is a weighted sum of intermolecular electrostatic (Elec), van der Waals (vdW), desolvation (Dsolv), and buried surface area (BSA) (22, 23). All complex conformations were shown and analyzed by VMD 1.9.32.

### Targeting Zebrafish

Target sequence of *MYH7* gene was selected by using the cloud-based informatics platform “Benchling” (https://www.benchling.com/crispr/). GRCz11 genome dataset was selected for determination of the target and guanine nucleotide was added to the 5′ of the target sequence to increase the efficiency of *in vitro* transcription. Template was generated by PCR using Phusion High-Fidelity DNA Polymerase (ThermoFisher Scientific) with the forward primer containing T7 promoter and targeted sgRNA sequence and the reverse primer encoding for the standard chimeric sgRNA scaffold (Supplementary Table 1). Scrambled (scr) gRNA was used as control. DNA template was purified with NucleoSpin Gel and PCR Clean up Kit (Macherey-Nagel GmbH & Co KG). sgRNAs were transcribed *in vitro* by using HiScribe™ T7 High Yield RNA Synthesis Kit (New England Biolabs). Transcription products were purified with RNA Clean & Concentrator-25 kit (Zymo Research, CA). RNA quantity was determined by NanoDrop™ OneC (Thermo Fischer Scientific).

### Ribonucleoprotein (RNP) Complex Preparation and Microinjection

sgRNA and Guide-it™ Recombinant Cas9 (Takara Bio Inc) were mixed at 1:1 (500/500 ng/μL) and incubated at room temperature for 5 min to generate the RNP complex. 0.05% phenol red was added to the mixture for visualization of injection. 1–1.5 nL of solution was injected directly into the cytoplasm of 1-cell stage zebrafish embryos.

### Zebrafish Genotyping

Embryos injected with RNPs were used for genotyping at 5 dpf. Ten larvae were transferred individually to 1.5 mL tubes in 50 μL DNA extraction buffer (10 mM Tris HCl pH:8.0, 50 mM KCI, 0.3% Tween 20, 1 mM EDTA) and incubated at 98°C for 10 min. Next, 3 μl of proteinase K solution (5 mg/mL; Sigma Aldrich) was added and incubated at 55°C overnight. Samples were incubated at 98°C for 10 min to inactivate proteinase K and centrifuged at 14.000 rpm for 4 min. Supernatants were transferred into new tubes, mixed with 50 μL ddH_2_O, 10 μL 3M sodium acetate, 180 μl isopropanol, vortexed and kept on ice for 10 min. Samples were centrifuged at 14,000 rpm for 15 min at 4°C. Supernatants were discarded and pellets were washed with ice-cold 70% ethanol and air-dried for 15 min. Pellets were resuspended in 10 μL nuclease-free water. Genomic DNA quantity was determined by NanoDrop™ OneC. Targeted *MYH7* loci that were amplified with PCR, used for Sanger sequencing. Genomic DNA was used as template at 100 ng/μL concentration and mixed with CloneAmp HiFi PCR Premix (Takara Bio Inc). Primers were designed to contain the mutation area (Supplementary Table 1). Final volume was adjusted to 25 μL with nuclease-free water. PCR was performed in SimpliAmp Thermal Cycler (Thermo Fischer Scientific) using the following conditions: 98°C for 1 min; 35 cycles of 98°C for 30 s, 62°C for 30 s and 72°C for 20 s; 72°C for 10 min. 1 μL of each sample was loaded into 1% agarose gel and electrophoresed. PCR products were cleaned up with NucleoSpin Gel and PCR Clean-up Kit. Products were quantified by using NanoDrop™ OneC.

### *In vivo* Imaging of Zebrafish Embryos and Larvae

Phenotypic evaluation of embryos and larvae was performed using an Olympus SZX2-ILLB stereomicroscope (Olympus Corporation, Japan). Images were captured with an Olympus SC50 microscope camera (Olympus Corporation, Japan).

### Whole-Mount *in situ* Hybridization

Larvae were collected at 3 dpf and 5dpf and fixed in 4% PFA for 48 h. Larvae were washed twice with PBT, twice with methanol for 5 min each at RT and stored at −20°C for further use. Antisense RNA probes for *MYH7, MYH7l, and mylk3* were synthesized. Briefly, a forward primer and two reverse primers were designed for each gene. One of the reverse primers were with T7 promoter sequence for *in vitro* transcription. First round of PCR was performed for amplification of the template. Second round of PCR was carried out to add T7 promoter. Probes were transcribed *in vitro* by using T7 RNA Polymerase (Thermo Fischer Scientific). Larvae were rehydrated by serial washes in 75% to 25% methanol/PBT for 5 min each followed by four PBT washes. Next, larvae were treated with Proteinase K (40 μg/μL) for 30 min, re-fixed in 4% PFA for 20 min and thoroughly washed afterwards. WMISH was performed as described previously (25, 26). Following prehybridization in hybridization buffer (Hyb) for 5 h at 67°C, samples were incubated with antisense probes in Hyb o/n at 67°C. The next morning, Hyb with probes were replaced with Hyb and incubated at 67°C for 20 min. Serial washes at 67°C were performed as follows: Three times in 2X 50% SSCT/50% formamide for 20 min, twice in 2X SSCT for 20 min and four times in 0.2X SSCT for 30 min. After a brief wash in PBT at RT, larvae were blocked with 5% sheep serum (Sigma-Aldrich) and 10 mg/ml BSA in PBT for 2 h and incubated in anti-Digoxigenin-AP Fab fragments o/n at 4°C (Merck&Co). After the removal of the antibody, larvae were washed in PBT over 3 h with 7–8 changes. Larvae were washed three times with NTMT buffer for 5 min, transferred into a 24 well-plate, stained with BCIP/NBT in NTMT buffer. Larvae were washed twice with PBT, incubated in stop solution for 1 h, washed twice with PBT and kept in 80% glycerol/20% stop solution until imaging (26, 27).

## Results

### Patients' Information

**Figure 1:**
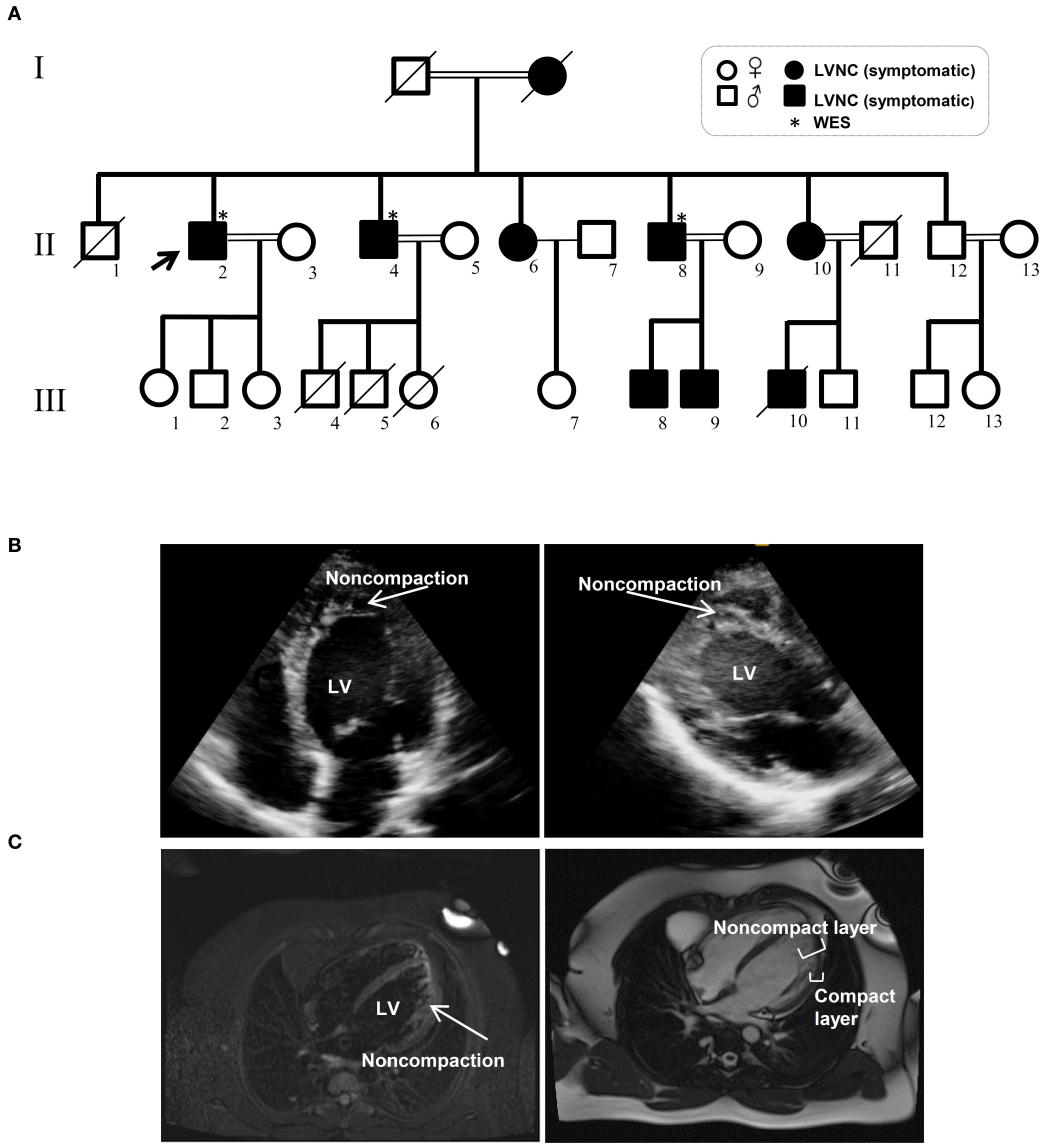
Pedigree and clinical manifestation of the left ventricular non-compaction (LVNC) in the affected members included in this study. **(A)** A pedigree of the family shows the number of affected members (affected members: shaded, WT: unshaded). A black arrow indicates the proband. Genotypes for the proband and all other family members are shown. **(B)** Echocardiograph (ECG) images of affected individuals with LVNC of the myocardium. **(C)** Magnetic resonance imaging (MRI) of thin epicardial and trabeculated endocardial layers of the ventricular wall.

The Iranian family examined in this study, consisted of individuals with an autosomal dominant inheritance pattern. The proband was 55 years old with diabetes mellitus and hypertension since age 34 due to multivessel nonsignificant coronary artery disease and moderate left ventricular dysfunction (LVEF: 35–40%). He received anti-ischemic therapy without any LVNC diagnosis. The second brother was 44 years old and had repeated non-angina chest pain since age 36. We recruited a 46-year-old brother as the third member, who was asymptomatic until age 37 when his symptoms started to manifest with dizziness and hypertension. Currently, he suffers from occasional non-anginal chest pain, intermittent dyspnea on exertion and peripheral edema. He is in New York Heart Association (NYHA) class II. The last brother, who is 35 years old (III-11), was examined twice and found to have a normal left ventricular wall without any signs of LVNC. All affected siblings had late onset symptoms of LVNC that occurred around the third decade of life. One offspring (III-10) died at the age of 12 with LVNC phenotype (Figure 1A and Table 1).

### Variant in Myosin Heavy Chain 7 (*MYH7*) Identified as a Candidate for LVNC

WES was performed using the Illumina NovaSeq platform, which generated paired-end reads of 150 base pairs with an average coverage of 100X for the three affected, well-characterized brothers (II-2, II-4, and II-8) who had clinically proven disease (Figures 1B,C and Table 1).

**Figure 2:**
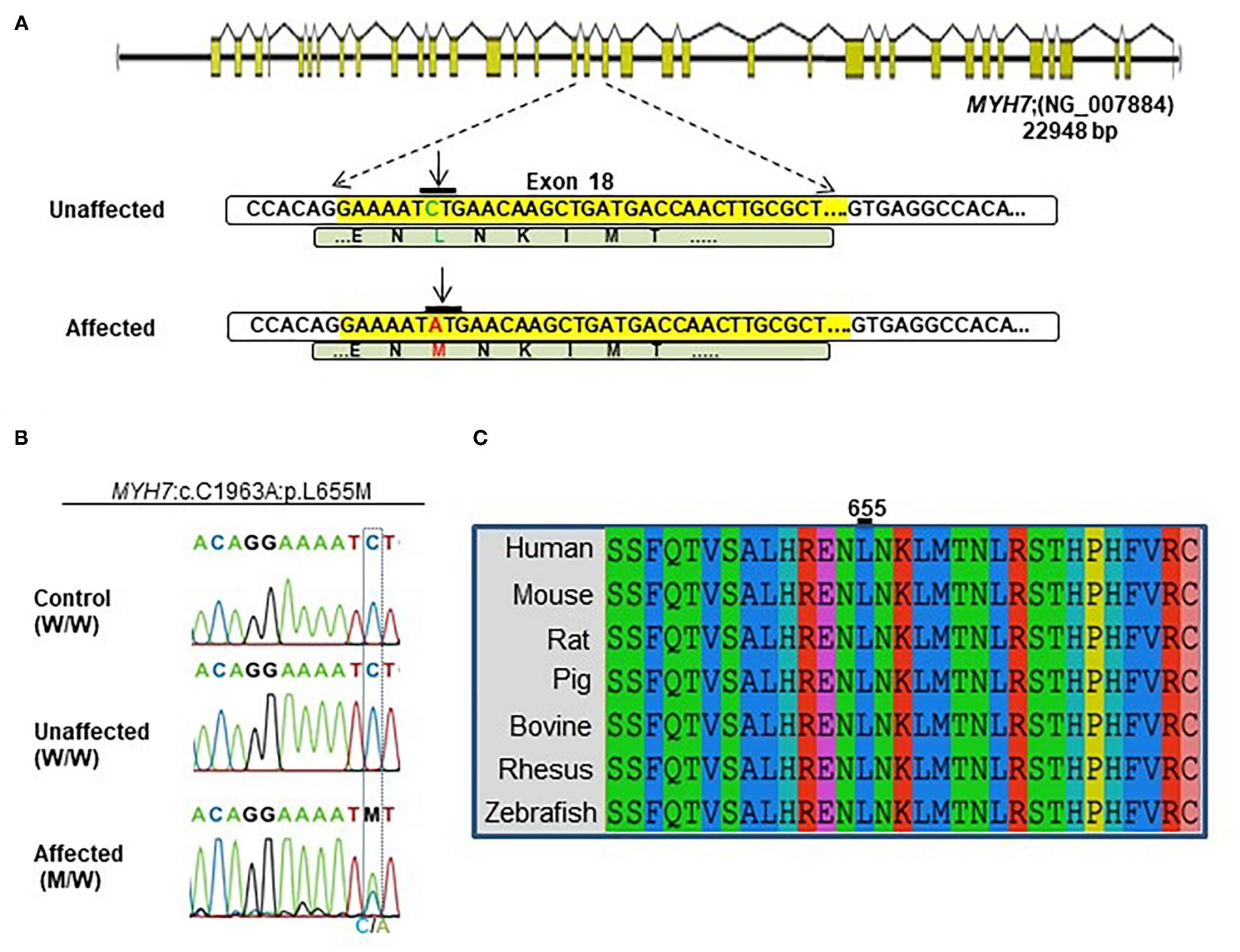
Identification of the *MYH7* heterozygous variant in the selected Iranian family. **(A)** Schematic diagram shows the human *MYH7* locus and the enlarged sequences of exon 18 of this locus, along with the sequences of the *MYH7* variation. The yellow region is the exon 18 sequences from *MYH7*. Green letters are wild-type DNA and the amino acid motif in the unaffected family members. Red letters indicate the substituted (C–A) target nucleotide in the *MYH7* and the p.Leu655Met alleles in the affected family members. **(B)** Sequence chromatograms of the *MYH7* novel variant loci which were found in this study. The results of Sanger sequencing from affected, unaffected, and control DNA samples confirmed that the variation was inherited from one of the parents in a heterozygous pattern. **(C)** Evolutionary conservation of *MYH7* in the actin-binding site domain shows that this region of protein is highly conserved throughout all species, especially in Leu655.

A novel non-synonymous variant in *MYH7* was identified (c.1963C>A:p.Leu655Met, GenBank: NM_000257) (Figure 2A). Myosin heavy chain beta (MHC-β) or *MYH7* protein is encoded by the *MYH7* gene on chromosome 14. The identified variant was a pathogenic missense variation. All three affected individuals were heterozygotes, which was further confirmed by Sanger sequencing (Figure 2B). Other variations in this gene are associated with HCM (13), DCM (28, 29) and LVNC (30–32). The resultant protein is composed of a myosin N-terminal SH3-like domain (residues 32–81), a myosin motor domain (residues 85–778) and an IQ domain (residues 781–810). *MYH7* is an actin based motor protein that exhibits ATPase activity (22, 24). On the other hand, this protein has two actin-binding regions (655–677 and 757–771) and one nucleotide-binding region (178–185) (22, 24).

Segregation analysis of all family members (20 members) confirmed the presence of heterozygous alleles (*MYH*7^W/M^) in all seven affected members and wild type alleles (*MYH*7^W/W^) within the healthy members (Figure 2B). *In silico* prediction tools were used for protein-protein interaction analysis to predict the functional consequences of p.Leu655Met at the structural level. Phylogenetically, this variant is located in a conserved region among mammalians, and zebrafish and belongs to the motor domain area of the *MYH7* protein (Figure 2C). Several prediction tools suggested this variant as a pathogenic one; MutationTaster2 predicted that it is a disease-causing mutation with a score of 0.489. SIFT showed a damaging score of 0.912 and Polymorphism Phenotyping v2 (PolyPhen-2) predicted a score of 0.971.

In preliminary studies, two boys in the third generation (III-8 and III-9) showed no specific clinical symptoms and appeared healthy at the initial consultation. However, after genotyping of all family members, it was found that III-8 and III-9 also carry this variation. To confirm the genotyping results, III-8 and III-9 were referred to a specialist, and they were diagnosed with mild LVNC symptoms.

### *MYH7* Expression Patterns

**Figure 3:**
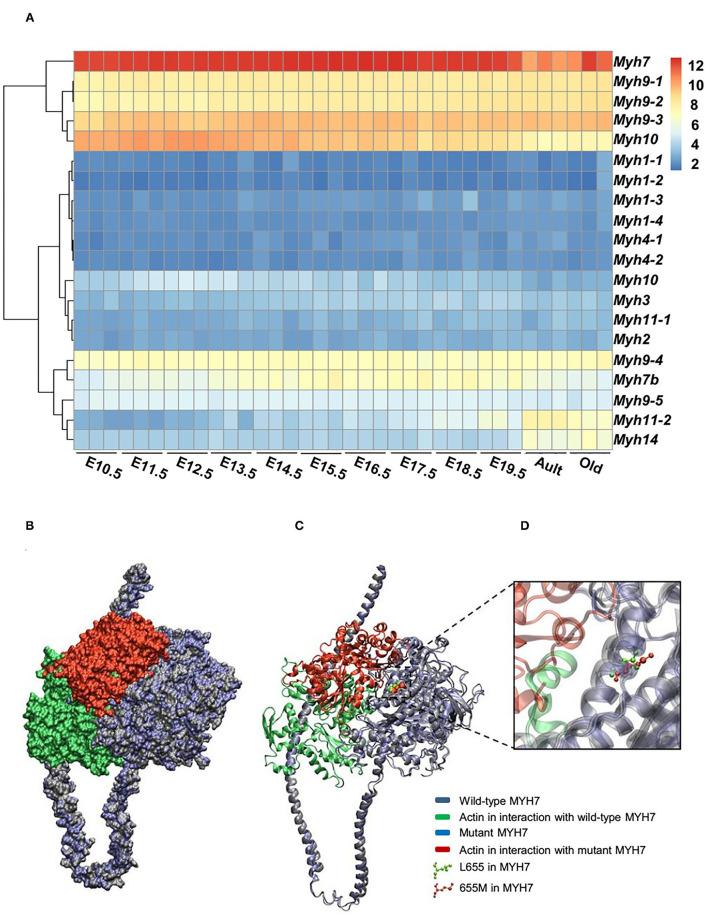
Expression patterns of all *MYH* superfamily members during cardiac development. **(A)** Differential expression pattern of *MYH7* in the mouse heart during 12 stages of development indicates that the expression of this gene plays an important role in the mouse cardiogenesis, especially during embryonic life. **(B)** Modeling scheme of the protein-protein interaction between native *MYH7*/actin and *MYH7* p.Leu655Met/actin at the surface. Actin in the wild-type position is shown in green and in the mutant position, is shown in red. **(C)** Secondary structure cartoon representation. Actin in the wild-type position is shown in green, and in the mutant position, is shown in red. **(D)** The Leu655Met mutation is highlighted. Wild *MYH7* protein (Leu 655) is shown in green, and the protein that has the mutated residue (Met 655) is shown in red.

The expression patterns of all myosin heavy chain classes were explored using a bio GPS database in order to investigate the tissue specificity of this protein. The results revealed high *MYH7* protein expression levels in the heart, skeletal muscle, and fetal thyroid (Supplementary Figure 1). This result further confirmed the phenotypic presentations of the affected members, which was cardiac dysfunction and mild muscle weakness. These data suggested that *MYH7* protein might be one of the major myosin heavy chain classes in the heart and skeletal muscle, and thus its mutations could result in cardiac and skeletal manifestations. Comparison of RNA sequencing data from various developmental stages of mouse heart revealed that, while all myosin classes were expressed, *MYH7* had the highest expression level in all embryonic stages as well as in the adult and aged mouse hearts (GEO: GSE93271) (Figure 3A). Furthermore, the *in silico* analysis revealed that various myosin classes are expressed during different cardiac developmental stages of zebrafish [at 24, 48, and 72 h post fertilization (hpf)] (GEO: GSE120236). And *MYH7* expression pattern showed the highest level among all myosin heavy chain classes in every stage (Supplementary Figure 2).

Contractile machinery of cardiac muscle is mainly dependent on actin and myosin proteins and their function; thus, it is likely that any structural associated dysfunction of these proteins would greatly affect cardiac performance.

### *In silico* Modeling for *MYH7*-Mutant Interactions With Actin

For evaluating the effect of the variation (p.Leu655Met) on *MYH7* function, the wild type and mutant minimized structures of *MYH7* were docked with actin through the HADDOCK2.2 web server. The most reliable complex conformation of the wild type and mutant *MYH7* interactions with actin were selected based on the HADDOCK2.2 results and presented here. Both complexes were aligned based on the *MYH7* structure and shown in surface (Figure 3B) and secondary structure cartoon (Figure 3C) representations. The averaged HADDOCK2.2 score over the top four members of the best cluster was −125.1 ± 13.7 for the wild type *MYH7*-actin complex, which increased for the mutant *MYH7*-actin complex (HADDOCK2.2 score = −115.1 ± 13.4). This result revealed that the mutant complex was relatively more unstable than the wild type complex. In addition, a comparison of the actin protein position in interaction with the mutant *MYH7* (red in Figures 3B–D) and wild type (green in Figures 3B–D) showed a mean root mean square deviation (RMSD value) of 48.05 Å, which indicated rotation and transition of this protein. In the p.Leu655Met mutation, a leucine amino acid is changed to methionine, which is a longer hydrophobic amino acid with an S-methyl thioether side chain. As it can be seen in Figure 3D, the amino acid at position 655 tends to the inner side of the protein. The methionine in this position would stabilize the structure with its longer hydrophobic side chain and S/π interactions between the side chain sulfur atom and aromatic amino acids. Thus, we postulate that this conformational change leads to a weaker binding energy of the actin protein to the 655M residue and subsequent functional insufficiency.

### Generation of *MYH7* Knockout Zebrafish

WES data revealed that p.Leu655Met point mutation in the *MYH7* gene is related with LVNC. Next, to investigate the role of *MYH7* in vertebrate heart development *in vivo*, we exploited clustered regularly interspaced short palindromic repeats/CRISPR associated protein 9 (CRISPR/Cas9) technology to generate a *MYH7* knockout (KO) zebrafish model (33, 34). We specifically designed a sgRNA to target exon 18, similar loci where the missense mutation occurred in patients. Following the microinjection of ribonucleoprotein (RNP) complex, we monitored the embryos daily under a stereomicroscope and observed a pronounced phenotype in majority of the embryos starting from 3 days post-fertilization (dpf). Next, we extracted genomic DNA from individual larvae at 5 dpf and proceeded with Sanger sequencing in order to determine insertions and deletions (indels). Sequencing results confirmed indels in *MYH7* gene with an efficiency of approximately 90% (data not shown). We further characterized *MYH7* KO zebrafish by using fluorescence microscopy to examine heart development and whole-mount *in situ* hybridization (WMISH) to detect mRNA expression of cardiac marker genes.

**Figure 4:**
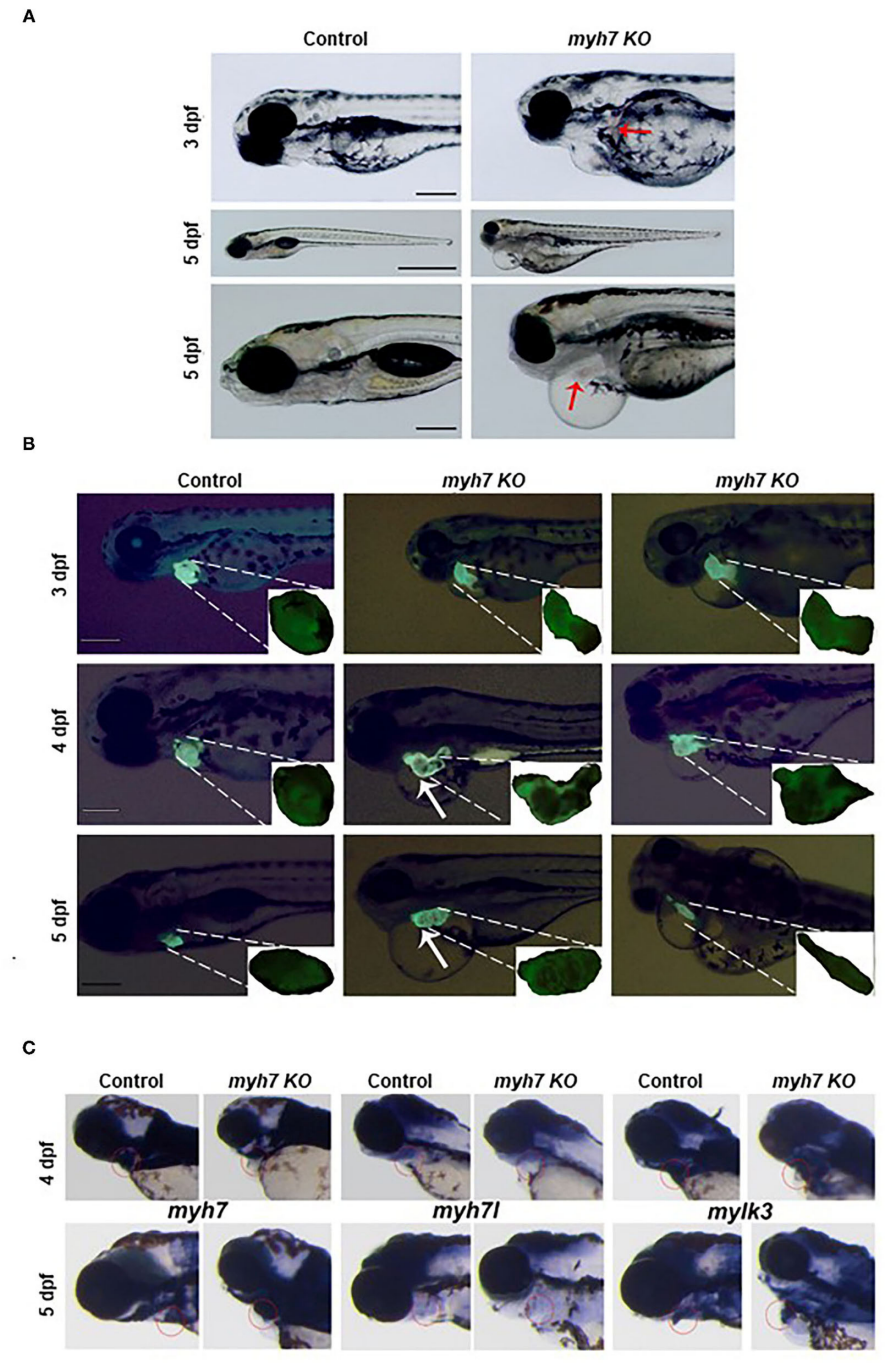
The *MYH7* deficiency causes cardiovascular defects in zebrafish. **(A)** Loss of *MYH7* disrupts heart development. Lateral view of 3 and 5 dpf scramble gRNA injected control group and *MYH7* KO larvae. Cardiac edema is clearly noticeable. Arrow at 3 dpf shows hemorrhage near heart. Edema accumulated around heart and spread to the abdominal area at 5 dpf. Arrow at 5 dpf shows enlarged heart. Scale bars: 200 μm in 3 and 5 dpf, 1 mm in middle 5 dpf. **(B)** The *MYH7* knockout zebrafish has distorted cardiac chamber formation and looping as shown by fluorescence imaging of the Tg(*actb2:hyper3*) transgenic zebrafish reporter injected with scramble or *MYH7* sgRNA. Representative images of control and mutant zebrafish are shown at 3, 4, and 5 dpf. Two different *MYH7* KO fish at 3 dpf showed abnormal looping. Two different *MYH7* KO fish at 4 dpf displayed enlarged and deformed ventricles. At 5 dpf, *myh*7 KO fish showed enlarged and granulated ventricle. Furthermore, a *MYH7* mutant zebrafish with tubular heart is shown. Scale bars: 200 μm. **(C)** *MYH7* is essential for proper formation of the cardiac ventricle and the pericardial cavity as shown by WMISH. Expression of *MYH7, MYH7l, and mylk3* mRNAs is strongly reduced in *MYH7* KO zebrafish at 4 dpf. *MYH7* expression expands to the pericardial cavity and ventricular chamber in *MYH7* KO fish at 5 dpf. *MYH7l* expression is reduced in *MYH7* KO fish at 5 dpf. Expression of *mylk3* in the cardiac ventricle of *MYH7* KO larvae is disrupted at 5 dpf.

Daily observation of *MYH7* KO zebrafish larvae revealed cardiovascular defects such as pericardial edema, chamber formation at pericardial sac and misshapen heart that became visible starting 3 dpf compared to the control sgRNA injected larvae. We also observed hemorrhage at various parts of the body near the heart and the vasculature system (Figure 4A). Pericardial and yolk sac/abdominal edema continued to expand until 5 dpf. At day five post fertilization, *MYH7* KO fish showed thickened heart with abnormal development of atrial and ventricular chambers as well as disturbed S-looping process (13), resulting in nearly unified heart (Figure 4A). Supplementary Video 1 shows a 5-dpf *MYH7* KO zebrafish larva with complications in heart contraction and blood flow, which are likely to be responsible for the hemorrhage. In addition, mutant fish exhibited reduced swimming ability and mobility (data not shown). Cardiac malformations advanced over time, where most of the larvae were dead at 7 dpf due to failure of proper cardiovascular system development. These data suggest that *MYH7* gene is essential for cardiovascular development.

The zebrafish heart has a tubular form at 1 dpf, later, it undergoes morphogenesis including looping and chamber ballooning at 2 dpf and forms the functional heart at 5 dpf (35). To understand how these processes are affected by *MYH7* loss-of-function, we injected *MYH7* sgRNA into the embryos of Tg(actb2:hyper3), a transgenic zebrafish reporter that contains the GFP driven by the *cmlc2* promoter (cmlc2:egfp) transgenesis marker within HyPer3 (36). The *MYH7* mutant zebrafish exhibited disorganized heart with enlarged atrium and ventricle along with abnormal looping at 3 dpf (Figure 4B). We observed several heart morphological defects in *MYH7* KO zebrafish. Majority of the larvae displayed elongated and granulated ventricle at 4 dpf (Figure 4B). At 5 dpf, loss of *MYH7* function was visible as bloated heart with thickened ventricle and altered S-looping formation (Figure 4B). Moreover, some larvae exhibited a tubular heart phenotype with visible separation between atrium and ventricle but no S-loop formation, resulting in a drastic reduction of the heart volume, ventricular contraction and blood flow rate (Figure 4B and Supplementary Video 2). These results indicate that *MYH7* is necessary for proper establishment of the heart at the morphological level and its contractile function.

### Cardiac Ventricular Gene Expression Is Disturbed in *MYH7* KO Zebrafish

Next, to investigate how *MYH7* mutation affected cardiac ventricular-related gene expression, we exploited WMISH by using the antisense RNA probes for *MYH7* and two other cardiac genes *myosin heavy chain 7-like* (*MYH7l*) and *myosin light chain kinase 3* (*mylk3*) on control and *MYH7* KO zebrafish larvae at 3 and 5 dpf. In control larvae, *MYH7, MYH7l*, and *mylk3* were properly expressed at the cardiac ventricle at 3 and 5 dpf (Figure 4C). In contrast, their expression levels were significantly reduced in *MYH7* KO larvae at 3 dpf. At 5 dpf, *MYH7* expression appeared to expand in parallel to the widening pericardial cavity and ventricular chamber (Figure 4C). The *MYH7l* was hardly detectable in the mutant larvae at 5 dpf. Expression of *mylk3* was also severely distorted in the cardiac ventricle of *MYH7* KO larvae, in accordance with its key role in enhancement of contraction force via regulation of actin-myosin interaction (Figure 4C) (37). These results suggest the *MYH7* role in heart development, especially in proper formation of the cardiac ventricle and the pericardial cavity.

## Discussion

*MYH7* or cardiac muscle beta isoform, the protein encoded by *MYH7*, is a member of the myosin superfamily of proteins expressed in muscle cells. *MYH7* contributes to movement and contraction, especially in cardiac cells, and plays a vital role in the sarcomere structure and heartbeat. Any mutation in *MHY7* can influence contractile properties and form-function relationship (32, 38) by affecting its binding to actin, MYBPC3, and TNNT2. The normal *MYH7* can also regulate ATP consumption and protein-protein interaction, and as a result, the binding regions of this protein and accompanying proteins are crucial to the function of myosin (39).

In this study, we used WES to elucidate the cause of LVNC in a highly affected Iranian family. WES analysis revealed a novel variation in the *MYH7* gene. Segregation analysis confirmed the variation in the affected members. In the next step, the expression pattern of myosin heavy chain classes was investigated in human tissues and in developmental stages of mice and zebrafish hearts. These analyses highlighted higher expression of *MYH7* in the heart and skeletal muscle, the entire mouse and zebrafish cardiogenesis, in addition to its presence in adult mice hearts. According to this finding, *MYH7* activity starts in the early embryonic stages of heart development. Due to its important role in the heart, its expression is distinct and excessive in this tissue. *MYH7* is also expressed differently from other myosin classes in the heart. A previous report also indicated higher expression levels of *MYH7* during the fetal stage of mice and its conditional depletion led to mild adulthood lethality (40, 41). The current RNAseq data analysis also suggested an important developmental role for myosins, especially for *MYH7*, as they are expressed ubiquistously during mouse cardiac organogenesis.

A novel missense variant was identified (c.1963C>A:p.Leu655Met) in the *MYH7* gene of a rare cardiomyopathy condition called LVNC by using WES analysis, a mutation that affected the *MYH7*-actin interaction. SIFT data showed that the region in which the new variant is located, is sensitive to amino acid changes and would impair *MYH7*-actin binding and its function. Thus, WES analysis provides great opportunity for researchers to find the correlation between the phenotype and genotype in inherited diseases. The results of *MYH7* protein alignment revealed that the original Leu655 is a conserved amino acid between several disciplines of vertebrates. These findings further highlight the importance of this protein's originality and the reason underlying its Leu655 conservation over the course of evolution. Although several researchers have investigated different variations in the vicinity of the actin-binding domain with the HCM phenotype (13, 42–44), This study suggested, for the first time, a novel variation of *MYH7* which might affect the actin-binding site that causes LVNC (45–47).

In mammalian muscles, actin-myosin machinery requires a regular cycle of ATP molecules and the frequent binding of two proteins, actin and myosin. This process is regulated by the myosin light chain protein (48, 49). Any malfunction of this regulator will reduce the contractile strength and loss of actin filament mobility (50, 51). The 3D analysis of myosin heavy chain classes by crystallography (52) and cryo-electron micrographs of actin-myosin complexes (53) provide platforms for studying this complex under different conditions (39). In the current study, we investigated the interaction between *MYH7* and actin at the new variation site. Three-dimensional modeling revealed that the *MYH7* mutant, which causes an amino acid exchange in the actin-binding site, resulted in a change in protein-protein interaction by actin rotation and transition. Interestingly, in other reports, similar variations near the actin-interacting domain were associated with different stages of HCM (13, 42, 54). However, in our affected family members, the pathogenicity is an LVNC phenotype, which is another type of cardiomyopathy. This difference between our clinical report and other studies needs to be investigated in more detail to find the appropriate correlation between these two phenotype manifestations.

An investigation to the effect of the uncovered variant(s) in terms of protein structure and function, may provide more information on the specific cellular events during an organ function as well as development. In the early stages of heart development, delamination of compact layer cardiomyocytes is the result of apical-basal polarization. Enrichment of the actomyosin network on the apical side of the delaminating cardiomyocytes has been reported (5). We suggest that the current *MHY7* variation may have altered the delamination process by affecting the frequency of apical-basal polarization and excessive response to tension heterogeneity. As a result, this variation induces more delamination signaling or more proliferation in delaminating cardiomyocytes, which eventually leads to irregular trabecular structures and deep recesses.

Here, we have generated the zebrafish knockout model of *MYH7* by using CRISPR/Cas9 genome editing technology and examined the influence of *MYH7* on cardiac morphogenesis and ventricular contraction. Cardiomyopathies have been modeled by using a variety of genetic or chemical manipulation techniques. The most abundant phenotypic indicators for zebrafish cardiomyopathy are (i) edema in the heart and the pericardial sac (ii) disrupted S-looping morphogenesis, and (iii) deformation of the heart anatomy. These phenotypic characteristics are consistent with those observed in other cardiomyopathy models (13, 55, 56).

Genetic screening of LVNC patients has identified several variations in genes encoding for sarcomeric proteins including *MYH7*, α-cardiac actin, cardiac troponin T, tafazzin, α-dystrobrevin, lamin A/C and dystrophin (30, 57, 58). Most of these genes have been associated with contractile function. Moreover, a mouse model of LVNC generated by a missense variation in the cardiac muscle gene *dystrobrevin-*α shows multiple trabeculations, expanded left ventricle and cardiac contractile dysfunction (59). Our *MYH7* KO zebrafish displays abnormally developed heart with pericardial edema, severe hemorrhage, distorted chambers and contraction problems. The correlation of features between these models suggest the use of our KO zebrafish as a platform to investigate the molecular mechanisms of *MYH7*-related disorder such as non-compaction cardiomyopathies.

The *MYH7* is major sarcomeric protein that is mainly expressed in the zebrafish heart. There are nine zebrafish homologs of the human *MYH* gene and seven of them share more than 80% protein homology with human *MYH7* (60). Being a component of the myosin complex, zebrafish *MYH7* is expressed in a variety of structures, including the heart primordium, the pericardial region and the musculature system during embryogenesis. The cardiac ventricle is a prominent structure where we detected high levels of *MYH7* in zebrafish embryos. Thus, disrupted heart formation and ultimate death of *MYH7* KO larvae suggests its key role in cardiovascular system development.

## Conclusion

In summary, this study introduced a novel genetic variation in *MYH7* (c.1963C>A:p.Leu655Met) and its corresponding clinical outcome was associated with LVNC. Data analysis and segregation study allowed us to predict new patients in the pedigree. Genotyping results combined with clinical phenotypes can confirm the function of any genetic variation and identify all affected, asymptomatic members in the pedigree for earlier and more effective treatment strategies. *In silico* and *in vivo* studies suggested actin-myosin binding changes as the underlying mechanism for *MYH7* variation phenotype.

## Data Availability Statement

The accession number for the variant c.C1963A reported in this paper is [ClinVar]: [SCV000886483.1]. The data presented in the study is available per request from corresponding author.

## Ethics Statement

The studies involving human participants were reviewed and approved by Royan Institute, Tehran, Iran. Written informed consent to participate in this study was provided by the participants' legal guardian/next of kin. The animal study was reviewed and approved by the Animal Experiments Local Ethics Committee of Izmir Biomedicine and Genome Center (IBG-AELEC). Written informed consent was obtained from the individual(s), and minor(s)' legal guardian/next of kin, for the publication of any potentially identifiable images or data included in this article.

## Author Contributions

MH, UB, SP, MB, and HB contributed to the conception and design of the study. SAM, NS, and MH performed bioinformatics analysis and functional analysis. MH and SP wrote the first draft of the manuscript. SJR, MT, and GO revised the manuscript. All authors contributed to the revisions and approved the submitted manuscript.

## Funding

This work was supported by a grant from Royan Institute, Tehran, Iran and the Scientific and Technological Research Council of Turkey (TUBITAK, grant no. 217Z123). GO Lab was funded by an EMBO Installation Grant (IG 3024).

## Conflict of Interest

The authors declare that the research was conducted in the absence of any commercial or financial relationships that could be construed as a potential conflict of interest.

## Publisher's Note

All claims expressed in this article are solely those of the authors and do not necessarily represent those of their affiliated organizations, or those of the publisher, the editors and the reviewers. Any product that may be evaluated in this article, or claim that may be made by its manufacturer, is not guaranteed or endorsed by the publisher.

## Supplementary Material

Supplementary Figure 1 Expression patterns of *MYH7* in different human tissues. Comparison of *MYH7* expression patterns in different tissues shows that this protein has elevated expression in skeletal and heart muscles.

Supplementary Figure 2 Expression patterns of *myh* superfamily members during zebrafish cardiac development in 24, 48, and 72 hpf.

Supplementary Table 1 Primer sequences for *MYH7* targeting sgRNA, scrambled control RNA, *MYH7* sequencing and RNA probes.

Supplementary Video 1 A 5 dpf *MYH7* KO zebrafish larva with complications in heart contraction and blood flow.

Supplementary Video 2 The *MYH7* KO zebrafish heart contraction and blood flow rate.
