# Supplementary figures and images for: A Novel Missense Variant in Actin Binding Domain of MYH7 Is Associated With Left Ventricular Noncompaction
Source: Front Cardiovasc Med. 2022 Apr 8;9:839862. doi: 10.3389/fcvm.2022.839862 (PMC9024299; doi:10.3389/fcvm.2022.839862)

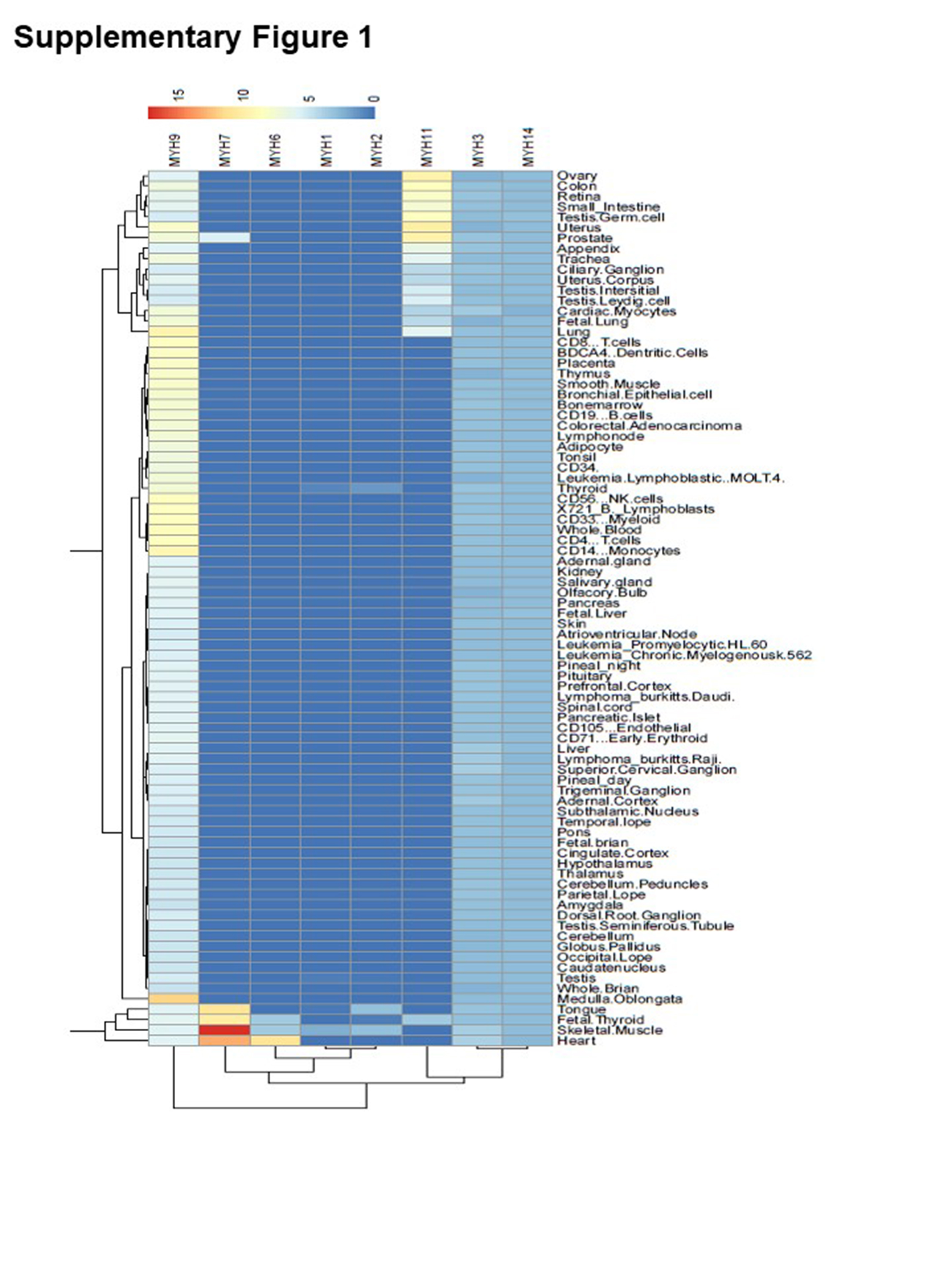

Supplement: Supplementary Figure 1 — Expression patterns of MYH7 in different human tissues. Comparison of MYH7 expression patterns in different tissues shows that this protein has elevated expression in skeletal and heart muscles. [file Image_1.JPG]

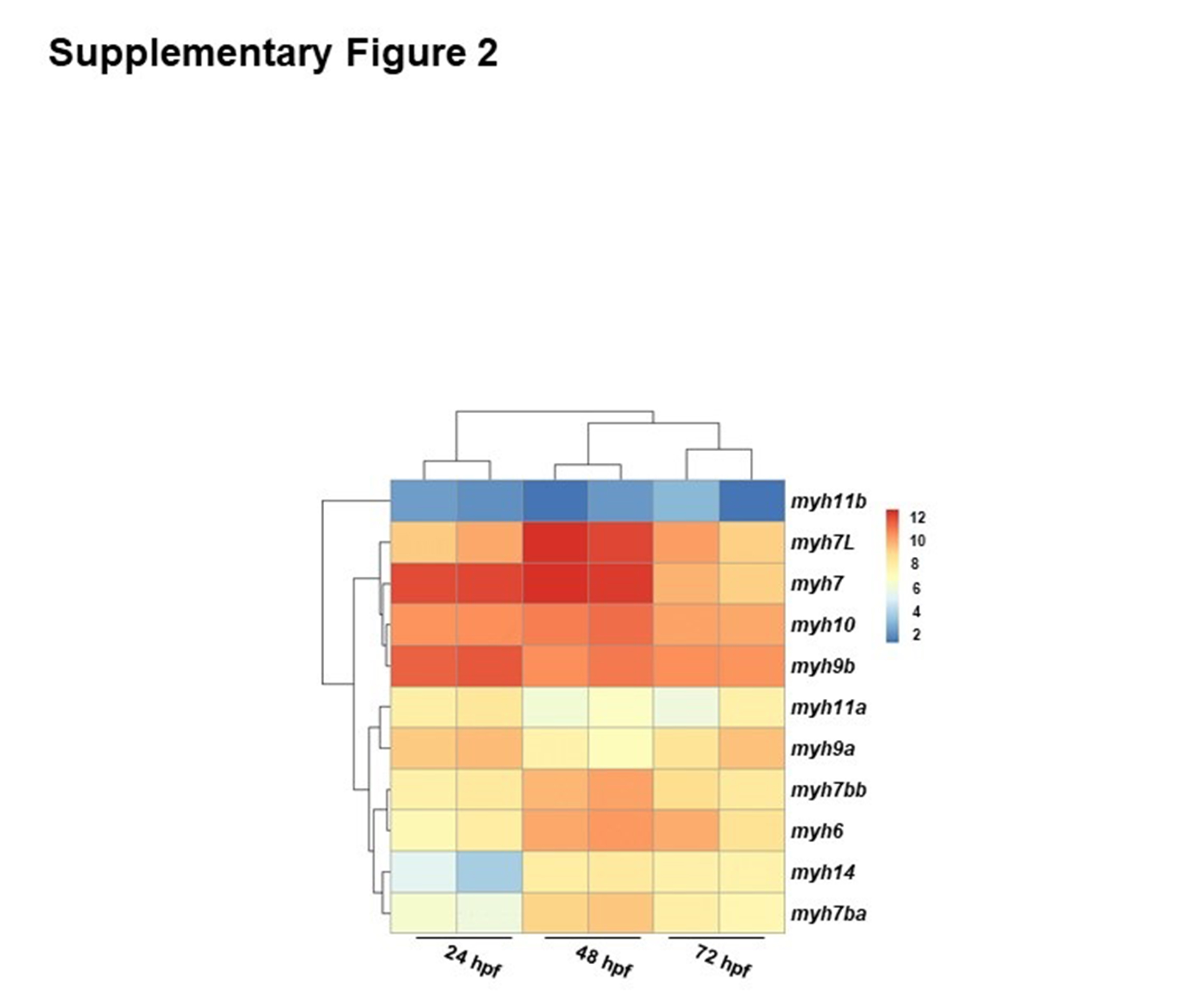

Supplement: Supplementary Figure 2 — Expression patterns of myh superfamily members during zebrafish cardiac development in 24, 48, and 72 hpf. [file Image_2.JPG]

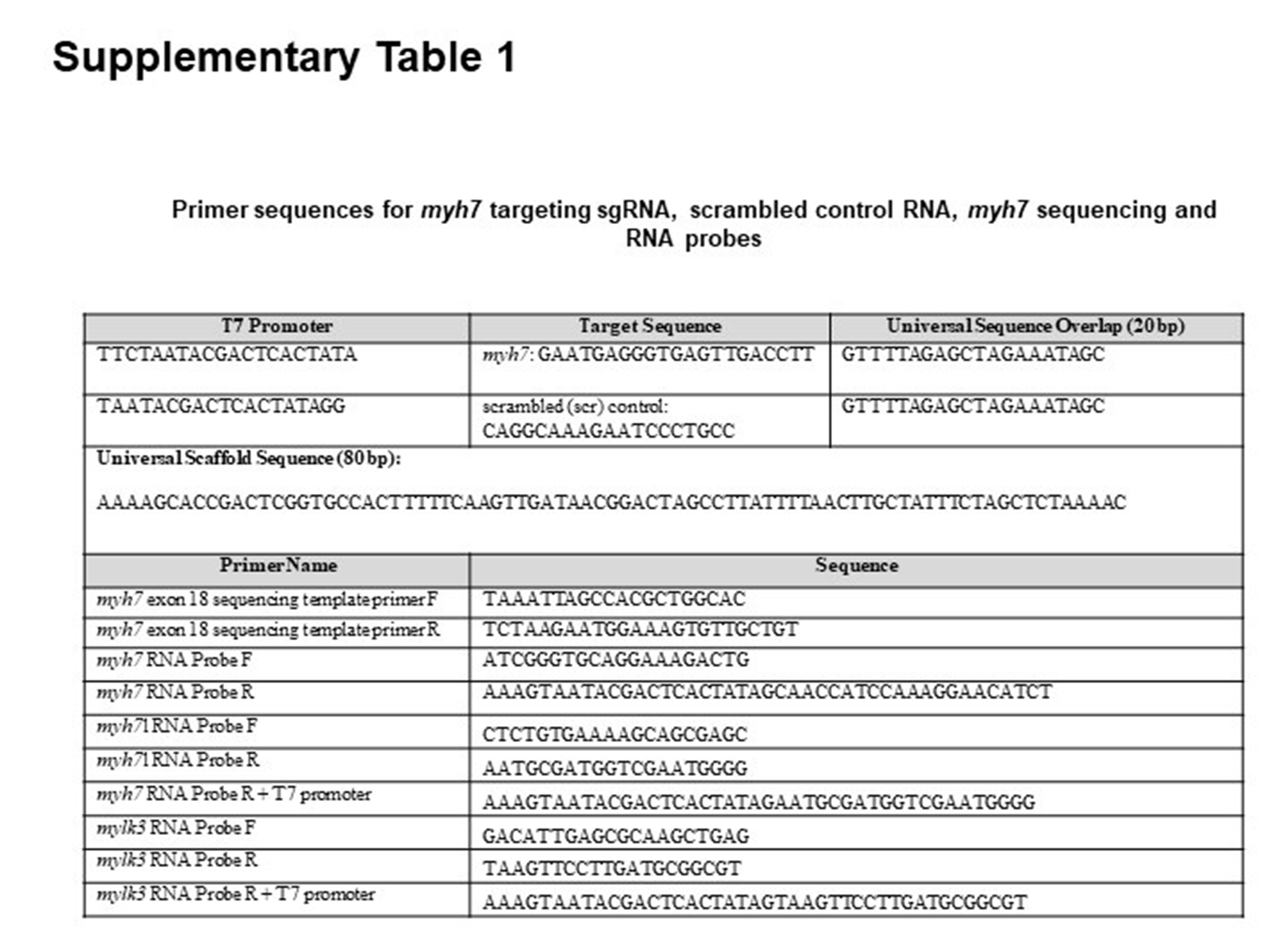

Supplement: Supplementary Table 1 — Primer sequences for MYH7 targeting sgRNA, scrambled control RNA, MYH7 sequencing and RNA probes. [file Image_3.JPG]
